# Colonization of fecal microbiota from patients with neonatal necrotizing enterocolitis exacerbates intestinal injury in germfree mice subjected to necrotizing enterocolitis-induction protocol via alterations in butyrate and regulatory T cells

**DOI:** 10.1186/s12967-021-03109-5

**Published:** 2021-12-18

**Authors:** Yu He, Weixia Du, Sa Xiao, Benhua Zeng, Xiang She, Dong Liu, Hua Du, Luquan Li, Fang Li, Qing Ai, Junli He, Chao Song, Hong Wei, Xiaodong Zhao, Jialin Yu

**Affiliations:** 1 Department of Pediatrics, Southern University of Science and Technology Hospital, Shenzhen, China; 2 Department of Neonatology, Children’s Hospital of Chongqing Medical University, Chongqing, China; 3 Shenzhen University Clinical Medical Academy, Shenzhen, China; 4 Chongqing Key Laboratory of Pediatrics, Chongqing, China; 5 Ministry of Education Key Laboratory of Child Development and Disorders, Chongqing, China; 6 National Clinical Research Center for Child Health and Disorder, Chongqing, China; 7 China International Science and Technology Cooperation Base of Child Development and Critical Disorders, Chongqing, China; 8 Department of Laboratory Animal Science, College of Basic Medical Sciences, Army Medical University, Chongqing, China; 9 Department of Neonatology, Shenzhen People’s Hospital, Shenzhen, China; 10 Precision Medicine Institute, The First Affiliated Hospital, Sun Yat-sen University, Guangzhou, China; 11 Department of Immunology, Children’s Hospital of Chongqing Medical University, Chongqing, China; 12 Children’s Hospital, Zhejiang University School of Medicine, National Clinical Research Center for Child Health, Hangzhou, China

**Keywords:** Necrotizing enterocolitis, Fecal microbiota transplantation, Gut microbiota, Germ free, Butyric acid

## Abstract

**Background:**

Necrotizing enterocolitis (NEC) remains a life-threatening disease in neonates**.** Numerous studies have shown a correlation between the intestinal microbiota and NEC, but the causal link remains unclear. This study aimed to demonstrate the causal role of gut microbiota in NEC and explore potential mechanisms involved.

**Methods:**

Eighty-one fecal samples from patients with NEC and eighty-one matched controls (matched to the NEC infants by gestational age, birth weight, date of birth, mode of delivery and feeding patterns) were collected. To explore if altered gut microbiota contributes to the pathogenesis of NEC, fecal microbiota transplantation (FMT) was carried out in germ-free (GF) mice prior to a NEC-induction protocol that included exposure to hypoxia and cold stress. Butyric acid was also administered to demonstrate its role in NEC. The fecal microbiota from patients and mice were analyzed by 16S rRNA gene sequencing analysis. Short chain fatty acid (SCFA) levels were measured by gas chromatography-mass spectrometry (GC–MS). The ontogeny of T cells and regulatory T cells (T_regs_) in lamina propria mononuclear cells (LPMC) from the ileum of patients and mice were isolated and analyzed by flow cytometry.The transcription of inflammatory cytokines was quantified by qRT-PCR.

**Results:**

NEC patients had increased Proteobacteria and decreased Firmicutes and Bacteroidetes compared to fecal control samples, and the level of butyric acid in the NEC group was lower than the control group. FMT in GF mice with samples from NEC patients achieved a higher histological injury scores when compared to mice that received FMT with control samples. Alterations in microbiota and butyrate levels were maintained in mice following FMT. The ratio of T_reg_/CD4^+^T (T_helper_) cells was reduced in both NEC patients and mice modeling NEC following FMT.

**Conclusions:**

The microbiota was found to have NEC and the microbial butyrate-T_reg_ axis was identified as a potential mechanism for the observed effects.

**Supplementary Information:**

The online version contains supplementary material available at 10.1186/s12967-021-03109-5.

## Introduction

Necrotizing enterocolitis (NEC) is a devastating gastrointestinal disease that occurs in neonates. It is one of the most common disease-related causes of death among low birth weight babies, and the mortality rate associated with NEC may be as high as 30% [1–3]. Survivors often experience severe complications such as short bowel syndrome and neurological sequelae that negatively impact quality of life [4, 5]. It is reported that hypoxemia, intestinal ischemia, immaturity, and other factors may trigger the inflammatory cascade that correlates with NEC [1]. In the last decade, the intestinal microbiota has been shown to play a role in NEC. Increased levels of Proteobacteria and decreased levels of Firmicutes were identified in NEC patients [6–8]. However, the direct link between the microbiota and NEC remains unknown. Causal relationships between the microbiota and host immunity can be strongly informed through the use of germ-free (GF) animal models, and have been widely used to study a variety of diseases such as obesity and depression [9, 10]. In this study, fecal microbial transplantation (FMT) was performed using GF mice to explore the direct link between fecal microbiota and NEC.

In addition, the underlying mechanisms concerning how the microbiota may give rise to NEC are unclear. Crosstalk between the microbiota and immune system can be altered during many microbial-related diseases [11, 12]. Short-chain fatty acids (SCFAs) are one type of microbial metabolite, which includes acetate, propionate, and butyrate, and are now a well-known link between the gut microbiota and host health [13]. A local immune modulating role of SCFAs has been clearly described and is known to induce the differentiation of regulatory T (T_reg_) cells in gut [14–16]. T_regs_ are a specialized subset of T cells, marked by the expression of the transcription factor fork head box protein 3 (Foxp3), which has a predominantly suppressive effect on many types of immune cells [17, 18]. Since an uncontrolled inflammatory response is a hallmark of NEC, it is reasonable to accept the limited evidence that has indicated that T_regs_ may take part in the pathogenesis in NEC [19, 20]. However, the specific role of T_regs_ in NEC patients remains unknown. In this study, we tested the hypothesis that the link between the microbiota and microbial metabolites and NEC is as least partially mediated by the regulation of T_regs._

In this limited study, the fecal microbiota and metabolites from NEC patients and controls were analyzed to identify how these factors influence NEC. Next, the change in T_regs_ in surgical NEC patients was analyzed to confirm the relationship between NEC and T_regs_. GF mice then received an FMT and underwent a NEC-induction protocol to demonstrate whether alterations in the gut microbiota play a role in the pathogenesis of NEC. Finally, the change in microbial communities and T_regs_ from ileal tissues of mice were assessed to explore the potential mechanism whereby a change in the microbiota and microbial metabolites influences the occurrence of NEC via their influence on T_regs_.

## Methods

### Patients and sample collection

This study was approved by the ethics committee of the Children’s Hospital of Chongqing Medical University, and collection of the clinical samples was carried out from January 2015 to October 2018. Informed consent was obtained from the parents of the neonates. All infants with definitive NEC, corresponding to Bell stage II–III, were included in the NEC cohort. Infants were control matched to the NEC infants by gestational age, birth weight, date of birth (± 2 months), mode of delivery and feeding patterns; we did not match the date of birth for surgical patients as we were unable to find appropriate matches for these cases [21]. Fecal samples from eighty-one NEC patients were collected by the medical staff once the diagnosis of NEC was complete, while the samples from eighty-one controls were collected at the same postnatal day as their matched NEC cases. Feces that were freshly evacuated into diapers were placed into sterile tubes and immediately transported to the laboratory. All samples were stored at – 80 ºC until further processing.

Tissue specimens from the ileum of nineteen surgical patients with NEC and nineteen controls (including patients with congenital intestinal atresia, spontaneous intestinal perforation, and re-anastomosis after meconium perforation repair) were obtained. The need for surgery was assessed by the pediatric surgeons. The tissue specimens were either treated immediately or stored at − 80 °C until needed for further analysis.

### DNA extraction and 16S rRNA gene sequencing analysis

DNA was extracted from frozen fecal samples using the QIAamp FAST DNA Stool Mini-Kit (Qiagen, Germany) according to the manufacturer’s instructions. Next, the DNA was amplified according to the V3-V4 region of the bacterial 16S rRNA gene with universal bacterial primers: 338F (5′-ACTCCTACGG-GAGGCAGCA-3′) and 806R (5′-GGACTACHVGGGTWTCTAAT-3′), which contained an eight-base barcode sequence unique to each sample. The purified PCR products were pooled in equimolar amounts and submitted for paired-end sequencing (2 × 250) on an Illumina MiSeq platform according to standard protocols. Raw FASTq files were quality-filtered by QIIME according to the index sequence. Reads with over one nucleotide mismatch in the primer sequence, ambiguous characters, a length shorter than 50 base pairs (bp), or those that could not be assembled were discarded. The sequences were binned into operational taxonomic units (OTUs) using similarity levels with a cutoff of 97% similar. The phylogenetic affiliation of each 16S rRNA gene sequence was analyzed using the Ribosomal Database Project.

### Mass spectrometry

SCFAs were detected and analyzed in the fecal samples using gas chromatography–mass spectrometry (GC–MS) technology (Thermo TRACE 1310-ISQ LT, America). Briefly, fecal pellets were homogenized in sterile deionized water (150 µL/sample) and centrifuged at 13,000 rpm, at 4 °C for 5 min, to pellet the particulate matter. A small sample of the supernatant (1 μL) was injected into the column and used for detection by GC–MS.

### Animal models and FMT

Fecal samples from randomly selected controls (n = 5, three from non-surgical patients and two from surgical patients) and NEC patients (n = 6, three from non-surgical patients and three from surgical patients) were used for FMT.The fecal samples for FMT were prepared according to slightly adopted methods from previous studies [22, 23]. All procedures were carried out under anaerobic conditions. Each fecal sample (100 mg) was suspended in reduced sterile phosphate buffered saline (PBS; 1.5 mL). Butyrate (100 mM) or pH-and sodium-matched PBS was intragastrically administered once at a volume of 0.02 mL/g body weight via silicone tubing (1.9fr) on the third day post-birth. FMT was performed on the 4th day post-birth to colonize the mice with fecal microbiota from either NEC patients or controls. Ten days after birth, the NEC model was established in the mice through exposure to hypoxia treatments (100% nitrogen gas for 1 min), followed by cold stress (4 ºC for 10 min) twice a day for four days according to a previous study [24]. All mice underwent establishment of the NEC model, and were randomly assigned into one of the following four groups: GN group (FMT with samples from NEC patients, n = 10) and GC group (FMT with samples from control patients, n = 10) were used for assessment of the NEC model in GF mice; the GNB group (pre-treat butyrate in mice receiving FMT with samples from NEC patients, n = 11) and GCB group (pre-treat butyrate in mice receiving FMT with samples from control patients, n = 11) were used to explore the causative role of the microbial product butyrate. Each group of mice was kept in separate isolators. Fecal samples were collected when the mice were killed on day 14 post-birth by using PBS (1 mL) to flush the large intestine. The enteric content was stored at – 80 °C until required for analysis by either GC–MS or 16SrRNA sequencing as described above.

### Tissue samples and isolation of lamina propria mononuclear cells

Ileal specimens from surgical patients or small intestinal tissue from mice modeling NEC were collected. Lamina propria mononuclear cells (LPMCs) were isolated as previously described [19]. Briefly, the tissue was washed in Hank’s balanced salt solution (HBSS) without Ca^2+^ or Mg^2+^ (Thermo, USA) and containing 5 mM EDTA (Sigma-Aldrich, USA). The residual lamina propria was digested in complete RPMI (Sigma-Aldrich) with collagenase type 1A (1 mg/mL; Sigma-Aldrich) for 1–2 h and then passed through a cell strainer (70 µm) to remove pieces of undigested tissue. LPMCs were washed using RPMI media and, following a slow-freeze, were stored in liquid nitrogen at a concentration of 10^6^ cells/mL with 10% fetal bovine serum (FBS). Another segment of tissue was stored in a solution containing 4% neutral buffered formalin. These tissue samples were subsequently paraffin-embedded and cut into 4 mm tissue slices, which were then stained with hematoxylin and eosin (H&E).The severity of NEC was evaluated by a researcher blinded to the experimental groups using a histological grading system [0 (normal ileum) to 4 (loss of intestinal villi with necrosis and transmural necrosis)] as previously described [25].

### Flow cytometry

Flow cytometry was performed using a 16-colour panel for flowcytometric analysis (BD, USA). Dead cells were excluded by staining with 7-aminoactinomycin D (7AAD; Biolegend, USA). LPMCs were stained with the following markers: CD4-FITC (fluorescein isothiocyanate; BD), CD3-PE-CY7 (phycoerthrin, cyanine 7; BD), CD25-APC (allophycocyanin; BD), and FoxP3-PE (eBioscience). After staining surface antigens, the cells were then fixed, permeabilized and stained for intracellularFoxp3 using the Foxp3 staining kit (eBioscience) according to the manufacturer’s instructions. The gating strategy was showing in Additional file 3: Table S3.

### Gene expression profile in tissues

Total RNA was extracted from 25 mg of fresh human NEC and congenital intestinal atresia ileumor mouse ileal tissue using the RNeasy Mini Kit (Qiagen, Germany). Total RNA was reverse transcribed using the PrimeScript™ RT reagent kit (TaKaRa, China). The reaction mixture containing the cDNA was added to each well of a 96-well PCR array for quantitative PCR using a Bio-Rad CFX96 Real-Time System (Bio-Rad, USA) to assess the levels of interleukin (IL)-1β, IL-6, IL-8,IL-10, tumor necrosis factor (TNF)-α and transforming growth factor (TGF)-β. The primer sequences are listed in Additional file 1: Table S1.

### Statistical analysis

All data were analyzed using SPSS version 22.0 software (SPSS Inc., USA). Data exhibiting a normal distribution were described as the mean with standard deviation (SD), and for comparison, an independent sample t-test or one-way analysis of variance (ANOVA) was applied. Data without normal distribution were expressed as the median (interquartile range) and analyzed using non-parametric tests. Proportions were compared using the χ^2^ or Fisher’s exact test, when appropriate. All statistical tests were two-sided and performed at a significance level of p < 0.05.

## Results

A total of 81 NEC patients and 81 control cases were included in this study. Both the NEC and control groups included 19 surgical patients. Non-NEC surgical patients included resections for spontaneous (focal) intestinal perforation (n = 5), tissue from re-anastomoses (n = 3), and congenital intestinal atresia (n = 11). The median birth weight and gestational age was similar between NEC and control groups in all patients, including the surgical and non-surgical groups. However, due to the nature of the non-NEC surgical diseases, we were unable to match the date of birth and days of the fecal sample collection, which resulted in a higher median age for sample collection in the NEC group. The patient characteristic data is summarized in Table 1.

**Table 1:** General information of patients with necrotising enterocolitis (NEC) and controls

	Total			Surgical patients		
	NEC group	Control group	p	NEC group	Control group	p
GA (weeks), median (IQR)	31.0 (29.4–33.7)	31.1(29.3–33.2)	0.75	31.8 (29.4–33.1)	33.3 (29.8–34.1)	0.26
Age (days), median (IQR)	15 (12–19)	15 (12–18)	0.81	11 (9–22)	4 (3–7)	< 0.01
BW (g), median (IQR)	1710 (1405–1970)	1720 (1360–1960)	0.84	1950 (1320–2230)	1920 (1670–2260)	0.62
Female, n (%)	40 (49.4%)	45 (55.6%)	0.43	10 (52.6%)	9 (47.4%)	0.75

*IQR* interquartile range, *NEC* necrotizing enterocolitis, *GA* gestational age, *BW* birth weight

### Change in microbiota and reduction of SCFA production in NEC patients

From the 162 samples, 7,011,868 high quality reads were identified with an average length of 436.2 base pairs (bps). The Venn diagram revealed that 2486 OTUs were shared between the NEC and control groups, while 1005 and 2991 OTUs were unique to NEC patients and controls, respectively.

**Fig. 1:**
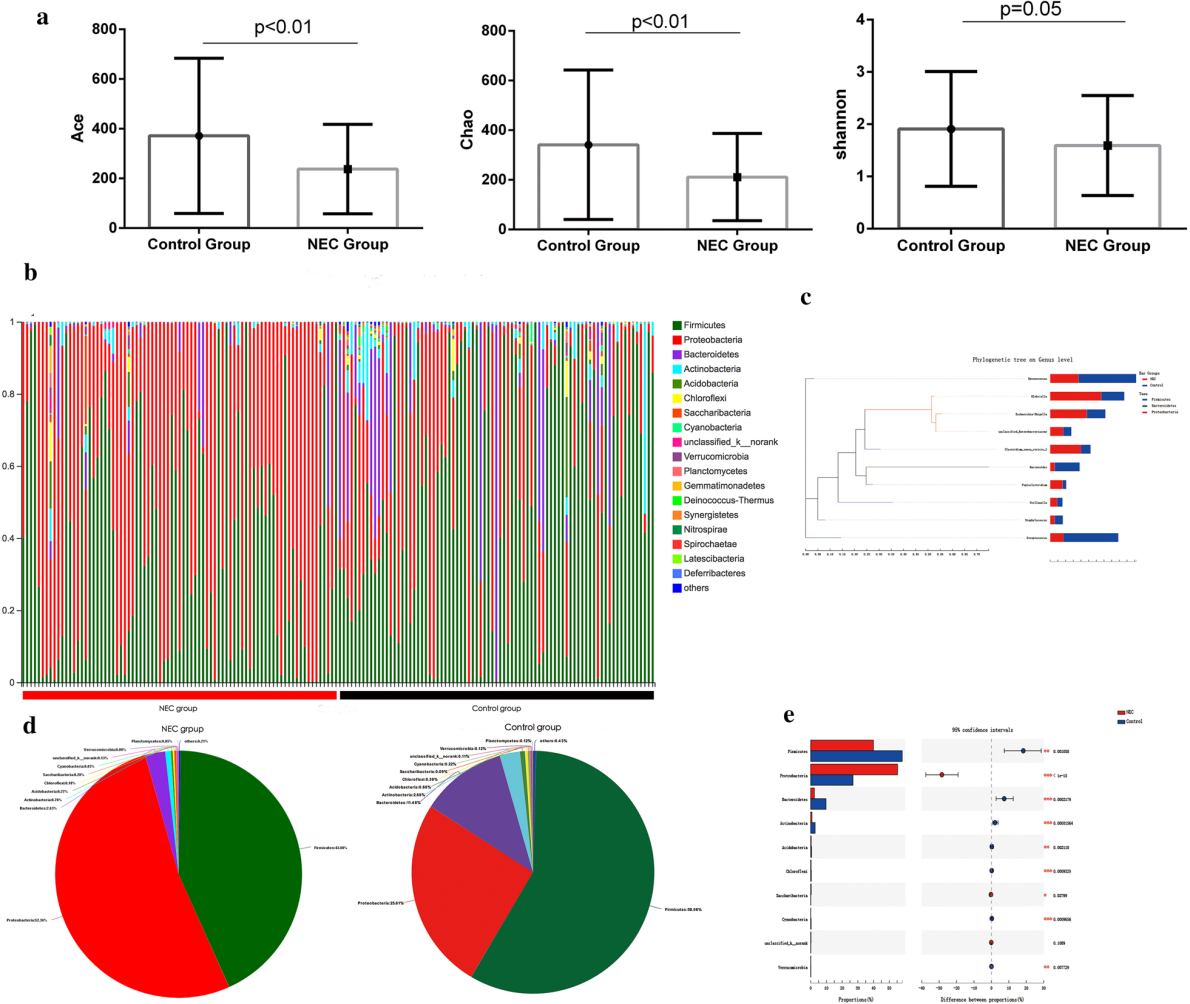
The intestinal microbiota in necrotizing enterocolitis (NEC) patients and control cases. **a** The α-phylogenetic diversity analysis revealed that NEC patients were characterized with lower richness in Ace, Chao and Shannon indexes compared to healthy controls. **b** Relative abundances at the phylum level present in each sample from NEC patients (left, red bar) and controls (right, black bar). **c** Phylogenetic tree of the 10 most abundant genera of gut microbiota in human NEC samples and controls. **d** The average relative abundances at the level of the phyla in the NEC and control groups. **e** Statistical analysis of the upregulated or downregulated phyla between the NEC and control groups

Next, the α-phylogenetic diversity indexes were analyzed to explore the community richness and diversity in two groups. Significant or near significant differences were evaluated for microbial richness and diversity. Compared to the control group, the NEC group showed decreased Shannon, Ace and Chao indexes, suggesting a decreased α-phylogenetic diversity in NEC patients (Fig. 1A). The overall microbial structure was then analyzed in each group at the phylum and genus levels. Proteobacteria, Firmicutes and Bacteroidetes were the most abundant bacteria in both groups and occupied over 90% of the total bacteria at the phylum level. The NEC group had a significantly higher proportion of Proteobacteria, with reduced proportions of Firmicutes and Bacteroidetes (p < 0.05, Fig. 1B, D, E). At the genus level, the 10 most abundant genera, including *Enterococcus, Klebsiella, Escherichia-Shigella,* and *Enterobacteriaccae*, belonged to either the Proteobacteria, Firmicutes or Bacteroidetes phyla (Fig. 1C).

**Fig. 2:**
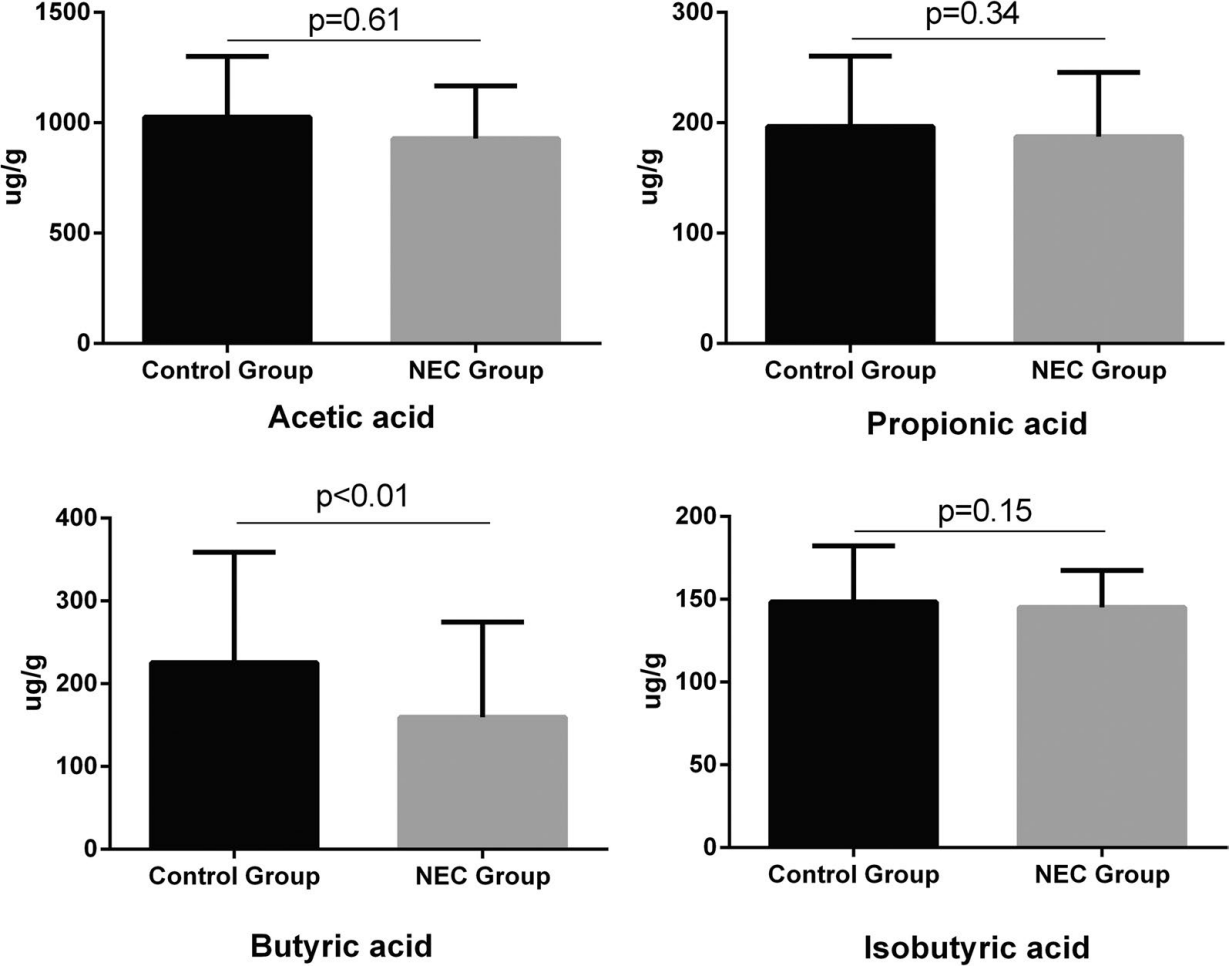
Gas chromatography–mass spectrometry (GC–MS) analysis of short chain fatty acids (SCFAs) in fecal samples from necrotizing enterocolitis (NEC) patients and control cases. Error bars indicate medianand interquartile range. The Mann–Whitney *U* test was used for comparisons between NEC and control groups.The level of butyrate was decreased in the NEC patients compared to the controls (p < 0.01)

Since SCFAs are known to be key mediators of inflammation, Phylogenetic Investigation of Communities by Reconstruction of Unobserved States (PICRUSt) was used for analysis and mapped to the Kyoto Encyclopedia of Genes and Genomes(KEGG) database to predict the potential function of the microbiota in each group according to previous studies [26, 27]. Importantly, the fatty acid biosynthesis pathway was found to be enriched in the control group (p = 0.01, Additional file 2: Table S2). Next, GC–MS was used to investigate the concentration of SCFAs in each sample. The results revealed that the levels of butyric acid were significantly lower in the NEC group compared to the control group (p < 0.01), while no significant differences were identified for acetic acid, propionic acid or isobutyric acid between the two groups (p > 0.05, Fig. 2). This result was similar to the functional analysis that showed the NEC group had increased enzymes for butyrate catabolism (p < 0.001, Additional file 1: Table S1).

### NEC patients exhibit significant alteration of T_reg_/T_helper_ cell ratio and related cytokines

**Fig. 3:**
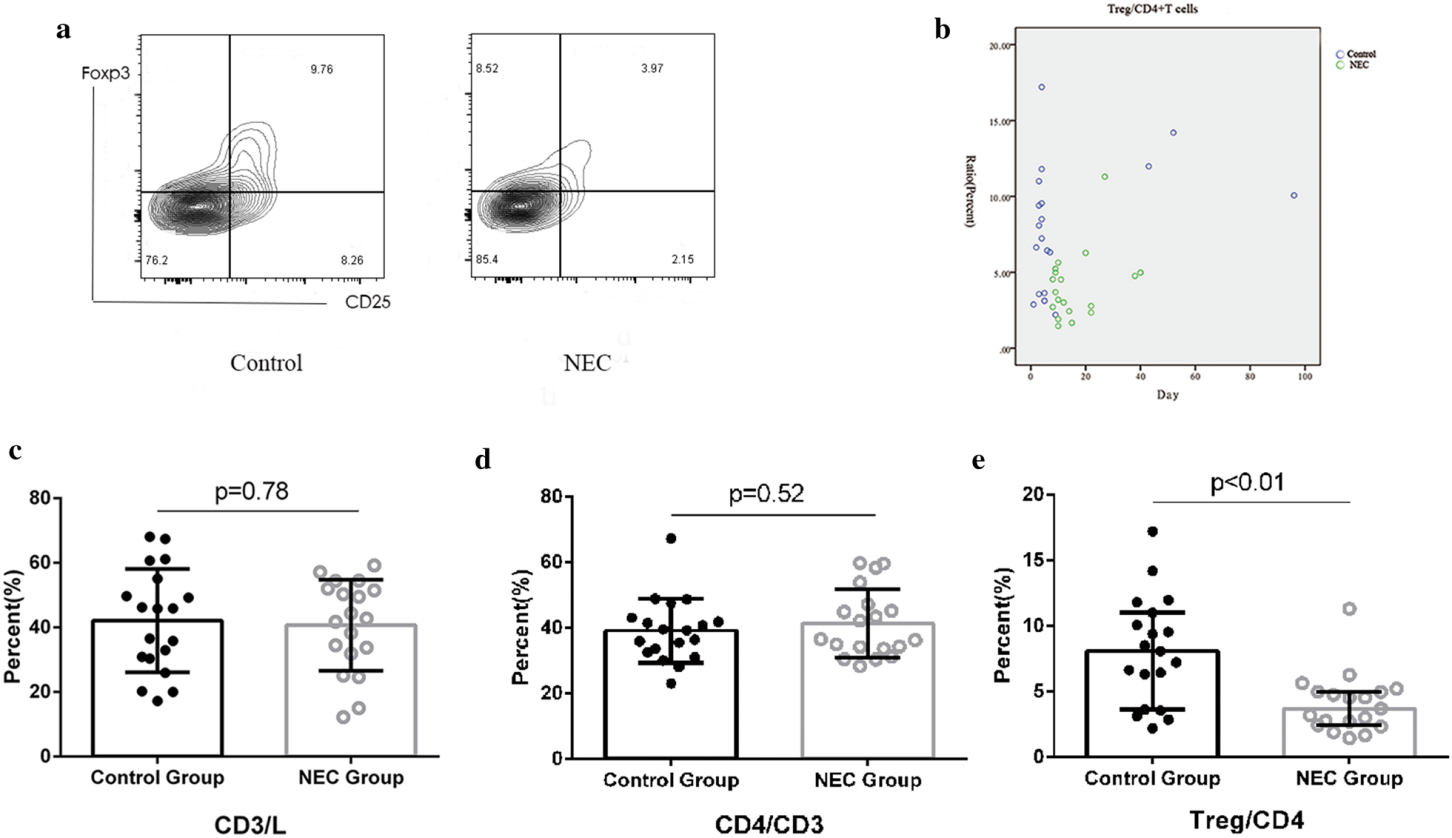
Proportion of lamina propria T_regs_ in surgical patients with necrotizing enterocolitis (NEC) versus non-NEC surgical controls. **a** Representative plot of the CD4^+^CD25^+^Foxp3^+^T cell population in surgical NEC and non-NEC surgical control patients. **b** Expression of lamina propria T_reg_/T_helper_ cell ratio by postnatal age. **c** Percentage of CD3^+^ T cells in the lymphocyte population from the ileal lamina propria of surgical NEC or non-NEC surgical control patients. **d** Percentage of T_helper_ cells in CD3^+^ T cells from ileal lamina propria of surgical NEC or non-NEC control patients. **e** T_reg_/T_helper_ cell ratio from ileal lamina propria cells of surgical NEC patients or non-NEC surgical control patients. Error bars indicate median and interquartile range. The Mann–Whitney *U* test was used for comparisons between NEC and control groups

The expression of T_reg_ cells in surgical patients was evaluated. The CD3^+^T cells/lymphocyte ratio and CD4^+^/CD3^+^T cell ratios did not differ between the two groups(p > 0.05, Fig. 3C). However, the proportion of T_reg_ (Foxp3^+^) cells compared with T_helper_ cells in the control group was significantly higher than that of the NEC group (medians were 8.08% vs 3.69%, respectively; p < 0.001). Since the surgical control patients had a significantly younger postnatal age, the possibility that postnatal age could influence the T_reg_/T_helper_ cell ratio was considered. Therefore, the relationship between the T_reg_/T_helper_ cell ratio and postnatal day, independent of surgical intervention, was explored and no significant correlation was identified (Fig. 3B).

**Fig. 4:**
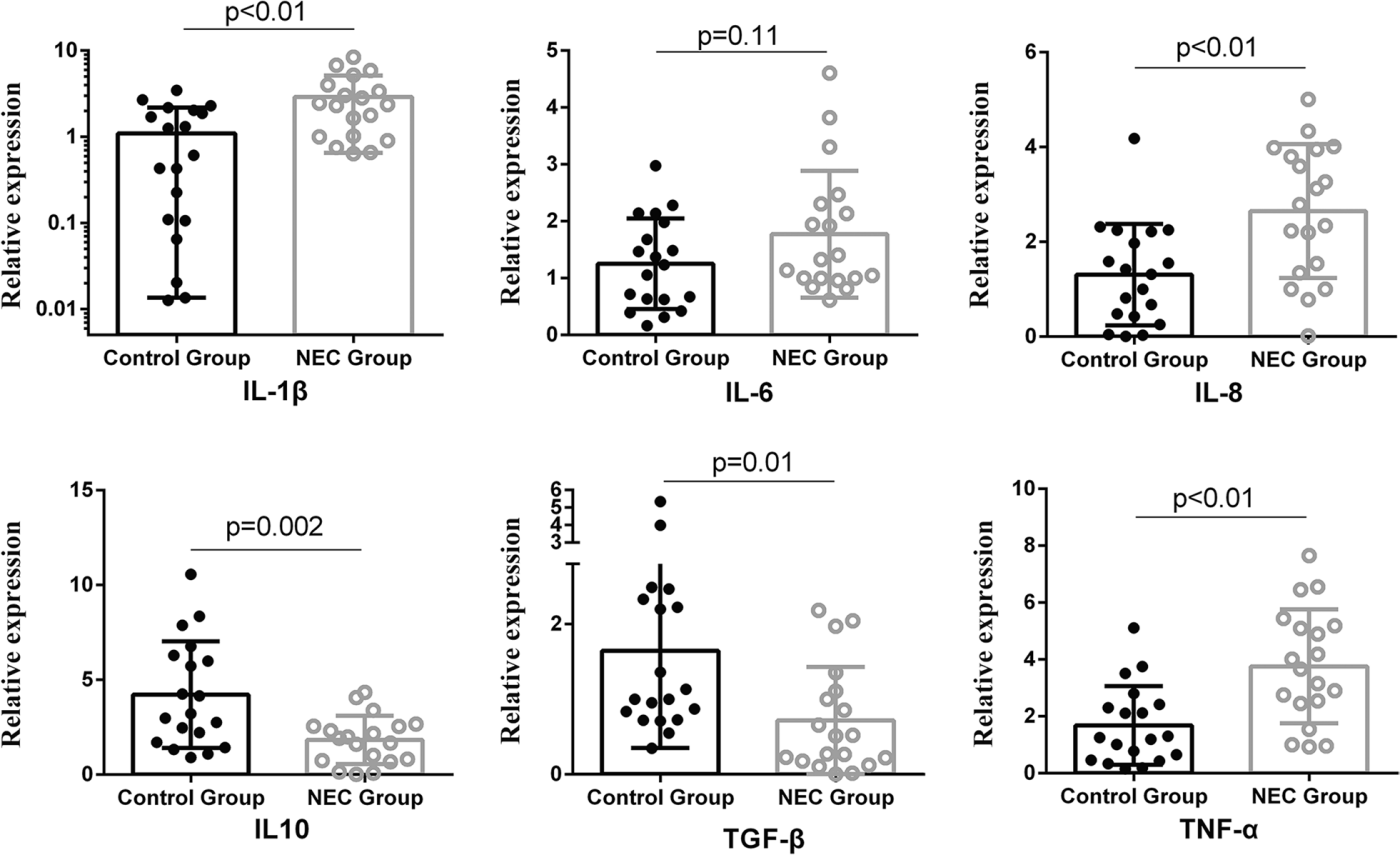
Cytokine gene expression in ileal tissues from surgical necrotizing enterocolitis (NEC) patients compared with non-NEC controls. *IL* interleukin, *TGF* transforming growth factor, *TNF* tumor necrosis factor

Cytokine expression profiles were also determined in both groups. Compared with surgical control cases, the surgical NEC cases showed significantly higher levels of IL-1β, IL-8, and TNF-α transcripts. NEC cases also had lower expression levels of IL-10 and TGF-β, which are related to the induction and function of T_reg_ cells (Fig. 4).

### Transplant of NEC patient microbiota into GF mice mimics NEC injury in the mice and alters the T_reg_/T_helper_ cell ratio

To demonstrate whether the microbiota of NEC patients directly contributes to the pathogenesis of NEC, FMT experiments were performed. GF mice were initially colonized with fecal microbiota from either NEC or control patients, and were then exposed to routine conditions used to establish the NEC model [28].

**Fig. 5:**
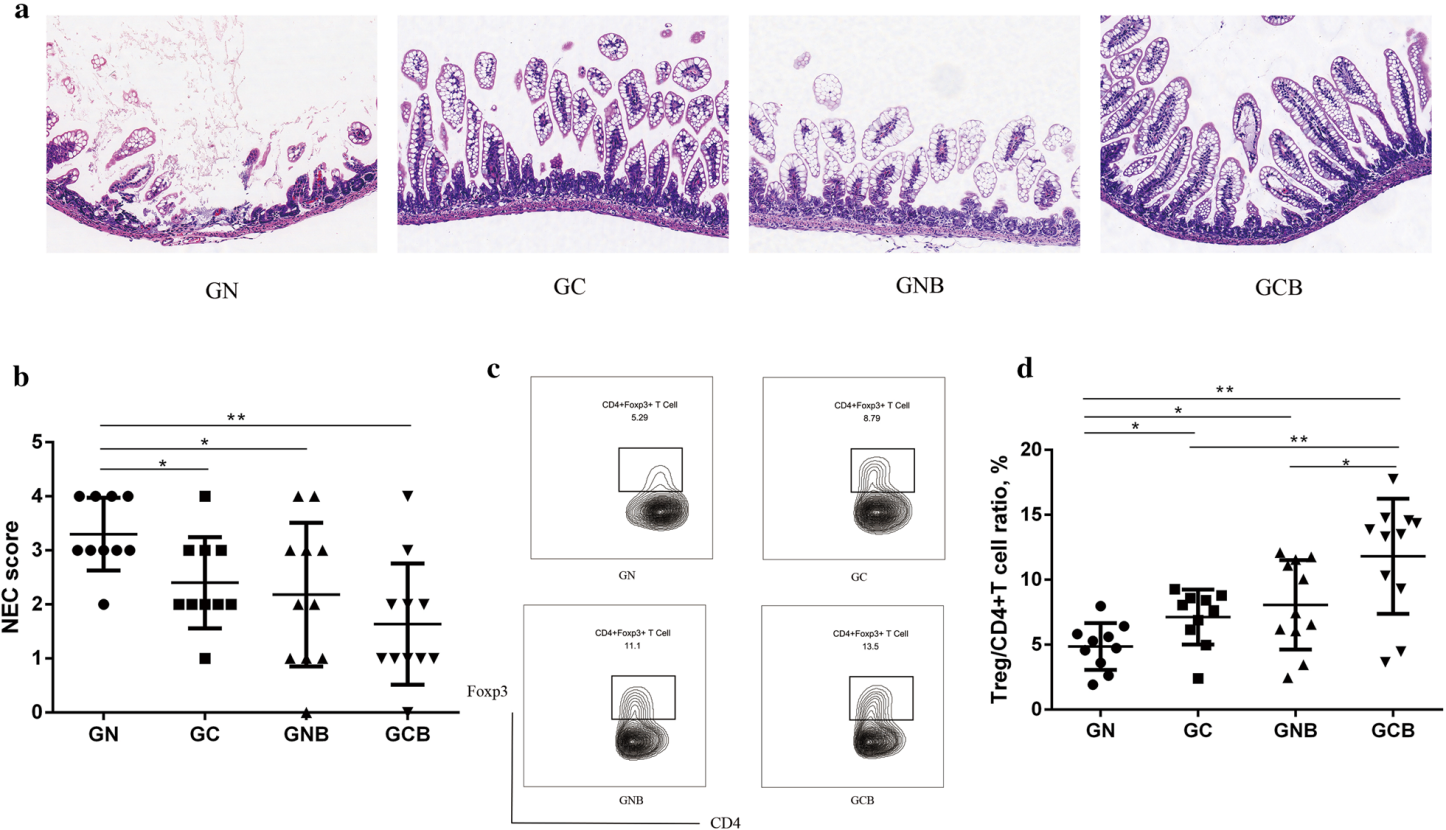
Comparative assessment of necrotizing enterocolitis (NEC)-like injuries and proportion of lamina propria T_regs_ between mice that received microbiota from NEC patients or control patients and underwent a NEC-induction protocol. **a** Histopathological evaluation of inflammation present in intestinal tissues from the GN, GC, GNB and GCB groups (100 × magnification). **b** NEC score of intestinal tissue injuries [0 (normal ileum) to 4 (loss of intestinal villi with necrosis and transmural necrosis)] observed in the GN, GC, GNB and GCB groups. **c** Representative flow plot of the T_reg_ population in the lamina propria of mice from GN, GC, GNB and GCB groups. **d** Percentage of T_reg_ cells in T_helper_ cells in lamina propria of mice from GN, GC, GNB and GCB groups. *GN* NEC patient fecal microbiota transplantation (FMT) plus NEC model, *GC* control patient FMT plus NEC model, *GNB* butyrate-treated with NEC patient FMT and NEC model, *GCB* butyrate-treated with control patient FMT and NEC model. GN, n = 10; GC, n = 10; GNB and GCB, n = 11. *p < 0.05; **p < 0.01

Histological analysis showed that the GN group suffered severe intestinal injuries (severe edema in submucosal and muscle layers, loss of villi with necrosis, and transmural necrosis) and had higher NEC scores when compared to the GC group (p < 0.05, Fig. 5A, B). These data indicated that colonization with the microbiota of NEC patients may contribute to NEC-like injury in mice. The number of T_reg_ cells was measured in the recipient mice, and a decreased T_reg_/T_helper_ cell ratio was identified in the GN group (p < 0.05, Fig. 5C, D), which was in accordance with the results from patient samples.

### Gut microbial alteration replicated in recipient mice

**Fig. 6:**
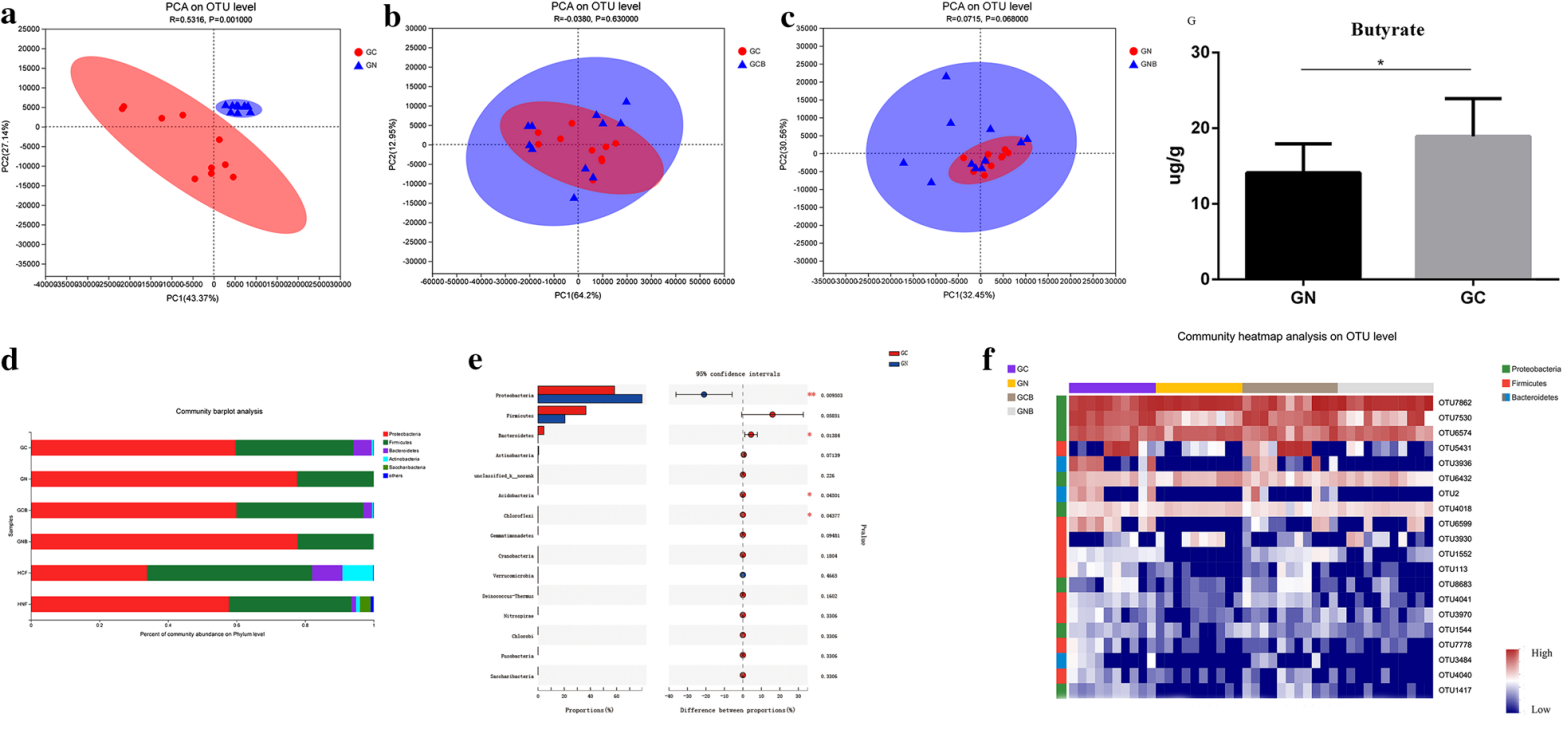
Gut microbial and metabolite characteristics of recipient mice after fecal microbiota transplantation (FMT). **a** At the operational taxonomic units (OTU) level, the principal component analysis (PCA) showed that gut microbiota composition of NEC microbiome recipient mice was significantly different compared to control microbiome recipient mice(p < 0.01). **b** No significant differences were found by PCA between the GC and GCB groups. **c** No significant differences were found by PCA between the GN and GNB groups. **d** The average relative abundances at the phyla level in each group. **e** Mann–Whitney *U* test of the upregulated or downregulated phyla in the GC and GN groups. **f** Relative abundance of top 20 OTUs responsible for discriminating NEC microbiome-recipient mice and control microbiome-recipient mice in GN, GC, GNB and GCB groups. **g** Level of butyrate in mice from GC and GN groups. GN, NEC patient fecal microbiota transplantation (FMT) plus NEC model; GC, control patient FMT plus NEC model; GNB, butyrate-treated with NEC patient FMT and NEC model; GCB, butyrate-treated with control patient FMT and NEC model; HCF, mixed fecal samples from control patients for FMT; HNF, mixed fecal samples from NEC patients for FMT. *p < 0.05

To determine whether the differences in the gut microbiome between NEC patients and healthy control cases could be reproduced in the recipient mice, the microbial communities in the intestinal content were harvested from each mouse, after the mice were killed, and subjected to 16S rRNA gene sequencing. Principal component analysis (PCA) revealed that the NEC mice had a distinct microbial pattern compared to the control mice (Fig. 6A). Importantly, the key characteristics that separated the gut microbiota observed in patients were also present in the recipient mice. The NEC mice had a higher level of Proteobacteria (p < 0.01) but lower levels of Firmicutes (p = 0.06) and Bacteroidetes (p < 0.05), which correlated with butyrate synthesis and mimicked the differences in microbiota found in NEC and control patients (Fig. 6D, E). Additionally, the top 20 differential OTUs responsible for discriminating between the GC and GN groups were part of the Firmicutes (9/20, 45%), Proteobacteria (8/20, 40%), or Bacteroides (3/20, 15%; Fig. 6F) phyla. To explore whether colonization with NEC microbiota could influence butyric acid synthesis in recipient mice, intestinal contents collected after the mice were killed were sent for GC–MS analysis. As shown in Fig. 6G, the concentration of butyrate in the GN group was lower compared to the GC group (p < 0.05).

### Replenishment of butyrate decreases the severity of NEC via the induction of T_regs_

To further verify the ability of butyrate to impact the pathogenesis of NEC, butyrate (100 mM) was administered to GF mice prior to the FMT and NEC modeling. Compared to the GN group, the GNB group had lower NEC scores (Fig. 5A, B). Butyrate also increased the T_reg_/T_helper_ cell ratio in both NEC FMT groups (GNB vs GN, p < 0.05) and control FMT groups (GCB vs GC, p < 0.01; Fig. 5C, D).

The composition of fecal microbiota among the GN, GNB, GC and GCB groups were also compared to determine if the effects of butyrate were dependent on a change in the gut microbiota. PCA revealed that the replenishment of butyrate did not significantly influence the microbial pattern in both NEC FMT groups (GN vs GBN; Fig. 6B) or control FMT groups (GC vs GCB; Fig. 6C). At both the phylum and OTU levels, a significant difference between the GN and GNB groups or GC and GCB groups was not observed (Fig. 6D, F).

## Discussion

In this limited study, the potential pathogenic role of the microbiota in NEC was demonstrated. Colonization of GF mice with fecal bacteria derived from NEC patients exacerbated NEC-like intestinal injuries in the mice after a NEC-induction procedure. The potential mechanism identified is the altered synthesis of butyric acid caused by a shift in the microbiota that leads to a reduction in T_reg_ cells.

It is widely accepted that the change of microbiota in early life correlates with the health of neonates and numerous studies have explored the relationship between the microbiome and NEC [6–8, 29–32]. Similar to most of these studies, we identified an increased proportion of Proteobacteria and decreased proportions of Firmicutes and Bacteroidetes in NEC patients. Based on this evidence, FMT was used to explore the microbiota as a potential pathogenic factor of NEC. Several novel studies have used FMT in NEC animal models [33–35]. However, none of these studies used GF mice. Additionally, FMTs were carried out after or during NEC modeling in these studies. Therefore, these studies mainly focus on whether FMT could be a potential therapeutic treatment for NEC. In the current study, GF mice were used to exclude the impact of the existing microbiota and the FMT was performed seven days before NEC modeling. Importantly, the altered composition of the microbiota and reduced concentration of butyrate in patients was reproduced in mice, suggesting that this strategy ensured the colonization of GF mice with the microbiota from the patients. In addition, the intestinal injuries common to patients were also maintained in mice, which indicated that colonization with the microbiota of NEC patients may contribute to NEC-like injury in mice and thus, confirmed the role of the microbiota and its metabolites in the pathogenesis of NEC. The association has been demonstrated previously between a high rate of Clostridium in NEC flora and a NEC outbreak in quails after FMT. Meanwhile, healthy premature infant fecal including high abundance of Bifidobacteria and no Clostridia was unable to produce NEC-like lesions [36]. However, the mechanism underlying how the change of the bacteria affect NEC occurrence had not been proved. The study had a small sample of infant feces, and due to technical limitations, only certain representative bacteria were analyzed, but now the development of second generation sequencing and metabolomic technologies present much more detailed picture to determine the mechanism of action of microflora in NEC.

SCFAs are a group of metabolites known as mediators of inflammation [13, 37, 38]. In the current study, butyric acid was lower in both NEC patients and FMT NEC mice compared to controls. Besides, replenishment of butyric acid could alleviate intestinal injury. A previous study has shown that breastfed pre-term infants had higher levels of fecal SCFAs compared with formula fed pre-term infants [39]. It is widely accepted that breastfeeding is a protective factor against NEC [1], and therefore these data support our results. The results of this study are also supported by another recent publication, which demonstrated that SCFAs are anti-inflammatory in immature human enterocytes [40]. Finally, previous studies have shown that most butyric acid is produced by bacteria belonging to either the Firmicutes or Bacteroidetes phyla, which also agreed with our results given that both NEC patients and FMT NEC mice had lower ratios of both Firmicutes and Bacteroidetes in their intestinal contents [41, 42].

Apart from exploring the role of the microbiota in NEC, the mechanism whereby the change in microbiota was able to influence the pathogenesis of NEC was investigated. A well-known function of butyrate in the modulation of local inflammation is the induction of T_reg_ cells [15, 16]. It is widely accepted that intestinal T_regs_ play a pivotal role in the suppression of immune responses. Effector T cells, such as T_h_17 cells, in addition to other myeloid cells and macrophages, contribute to intestinal inflammation and tissue damage in NEC patients. Therefore, the immune system within the intestinal mucosa needs to be strictly regulated, and T_regs_ likely play a major role in these counterbalance mechanisms [24, 43–46]. In our study, we found that the T_reg_/T_helper_ cell ratio decreased in both NEC patients and FMT NEC mice independent of the postnatal age since the postnatal days in the surgical patients are not likely to influence the comparison between the two groups, which were in accordance with previous studies [19, 44].

Butyrate is able to induce T_regs_ through several known mechanisms. Known as a histone deacetylase (HDAC) inhibitor, butyrate can promote the acetylation of histones at the Foxp3 promoter or the enhancer sequence conserved noncoding DNA sequences (CNS)1 and CNS3 to enhance T_reg_ induction [14, 15]. In addition, signaling mediated by G-protein coupled receptors (GPRs) for butyric acid stimulates T_reg_ induction. Biding of GPR109 by butyrate can promote the expression of IL-10 and aldehyde dehydrogenase in macrophages and dendritic cells, which also induces the generation of T_regs_ [37]. Moreover, butyrate can directly stimulate the proliferation of T_regs_ through GPR43 [16]. In our FMT-driven murine model of NEC, the administration of butyric acid could reduce intestinal inflammation, possibly via the induction of the induction of T_regs_, indicating that butyrate might act as the bridge between microbial metabolites and intestinal damage in NEC.

There are some limitations to this study. Firstly, metagenomics were not used to describe the composition of the microbiota. This was because the amount of microbial DNA required for metagenomics exceeded the volume of some of the human samples we were able to collect in the early stages of this research, and it also exceeds the amount of sample we can collect from the mouse model. To the best of our knowledge, very few studies have used metagenomics to explore the microbiota in NEC patients, and none have used it this way in mice [47, 48]. Alternatively, we used a functional inference analysis through a database with metagenomic data, using gene markers and reference genomes to help identify the differential metabolic pathways on which we focused. Since GC–MS was also used to directly detect the metabolites, the absence of metagenomic data may not influence the reliability of the results. Secondly, we did not distinguish between thymus derived T_regs_ (tT_reg_) and peripherally derived T_regs_ (pT_reg_). However, we did attempt to use Helios-BV421 to stain the T_reg_ population and differentiate between T_reg_ and pT_reg_ cells, but we were limited by the small numbers of T_reg_ cells in newborn mice. Since these data may not be reliable, we did not include and interpret these results. Based on previous studies that have revealed over 80% of T_regs_ in the small intestinal and colonic lamina propria to be pT_reg_ cells, and in most cases, microbial metabolites such as SCFAs can promote the differentiation of pT_regs_ and maintain proliferation of tT_regs_, we believe that this limitation did not influence our conclusions [18, 49]. Thirdly, although the gestational ages were matched, the age since birth of the surgical NEC patients was not matched to surgical controls since the timing of the surgeries in control patients necessarily differed from NEC patients. However, these data did not affect the T_reg_/T_helper_ cell ratio in our study.

## Conclusions

In this limited study, the relationship between the gut microbiota and NEC was demonstrated using FMT in GF mice. We found that an underlying mechanism of microbiota-driven NEC may be the decreased level of microbial derived butyrate, which in turn results in a diminished number of Tregs. Since Tregs play a key role in regulating inflammation, their reduction may contribute to the initiation of NEC. These findings provide a clear link between the microbiota and NEC, and present novel microbial metabolites to explore for the prevention of NEC.

## Supplementary Information

**Additional file 1**: **Table S1**. Primers of the cytokines.

**Additional file 2**: **Table S2**. Predicted functional differences in fatty acid metabolism between necrotizing enterocolitis (NEC) and control fecal samples.

**Additional file 3**: **Table S3**. Gating strategy for flow cytometry.

## Data Availability

Data generated or used in this study are available from the corresponding authors upon reasonable request.

## Abbreviations

NEC: Necrotizing enterocolitis
GC–MS: Gas chromatograph–mass spectrometer
FMT: Fecal microbiota transplantation
GF: Germ free
LPMC: Lamina propria mononuclear cells
IQR: Interquartile range
Treg: Regulatory T cell
PICRUSt: Phylogenetic Investigation of Communities by Reconstruction of Unobserved States
KEGG: Kyoto Encyclopedia of Genes and Geomes
