# Supplementary material for: Colonization of fecal microbiota from patients with neonatal necrotizing enterocolitis exacerbates intestinal injury in germfree mice subjected to necrotizing enterocolitis-induction protocol via alterations in butyrate and regulatory T cells
Source: J Transl Med. 2021 Dec 18;19:510. doi: 10.1186/s12967-021-03109-5 (PMC8684079; doi:10.1186/s12967-021-03109-5)
Supplement: Supplementary file 1 — Additional file 1: Table S1. Primers of the cytokines. [file 12967_2021_3109_MOESM1_ESM.doc]

| Table S1 **Primers of the cytokines** | |
| --- | --- |
| Primer | Sequence |
| Actin-F | GTGAAGGTGACAGCAGTCGGTT |
| Actin -R | GAGAAGTGGGGTGGCTTTTAGGA |
| Human IL-10 -F | TGGGGGAGAACCTGAAGA |
| Human IL-10 -R | ATGGCTTTGTAGATGCCTTTC |
| Human TGF- beta-F | CTGGCGATACCTCAGCAAC |
| Human TGF- beta -R | TAAGGCGAAAGCCCTCAAT |
| Human IL1b -F | TGGAGCAACAAGTGGTGT |
| Human IL1b -R | TTGGGATCTACACTCTCCAGC |
| Human IL6 -F | TCTCCACAAGCGCCTTCG |
| Human IL6 -R | CTCAGGGCTGAGATGCCG |
| Human IL8 -F | AACTTCTCCACAACCCTCTG |
| Human IL8 - R | TTGGCAGCCTTCCTGATTTC |
| Human TNF- α-F | CGAGTGACAAGCCTGTAGC |
| Human TNF- α -R | GGTGTGGGTGAGGAGCACAT |
