# Supplementary material for: Colonization of fecal microbiota from patients with neonatal necrotizing enterocolitis exacerbates intestinal injury in germfree mice subjected to necrotizing enterocolitis-induction protocol via alterations in butyrate and regulatory T cells
Source: J Transl Med. 2021 Dec 18;19:510. doi: 10.1186/s12967-021-03109-5 (PMC8684079; doi:10.1186/s12967-021-03109-5)
Supplement: Supplementary file 2 — Additional file 2: Table S2. Predicted functional differences in fatty acid metabolism between necrotizing enterocolitis (NEC) and control fecal samples. [file 12967_2021_3109_MOESM2_ESM.doc]

| Table S2. Predicted functional differences in fatty acid metabolism between necrotizing enterocolitis (NEC) and control fecal samples. | | |
| --- | --- | --- |
| Pathway | Fold ratio NEC/Control | P Value |
| Butyrate catabolism enzymes | 1.152 | <0.001 |
| Fatty acid biosynthesis | 0.898 | 0.01 |
