# Supplementary material for: Colonization of fecal microbiota from patients with neonatal necrotizing enterocolitis exacerbates intestinal injury in germfree mice subjected to necrotizing enterocolitis-induction protocol via alterations in butyrate and regulatory T cells
Source: J Transl Med. 2021 Dec 18;19:510. doi: 10.1186/s12967-021-03109-5 (PMC8684079; doi:10.1186/s12967-021-03109-5)
Supplement: Supplementary file 3 — Additional file 3: Table S3. Gating strategy for flow cytometry. [file 12967_2021_3109_MOESM3_ESM.doc]

| Table S3 **gating strategy for flow cytometry** | |
| --- | --- |
| Cell population | Gating strategy |
| Human tissues |  |
| T cell | 7AAD- CD3+ |
| CD4^+^ T cell | 7AAD- CD3+ CD4+ |
| Treg cell | 7AAD- CD3+ CD4+ CD25 high Foxp3+ |
| Mice tissues |  |
| T cell | 7AAD- CD3+ |
| CD4^+^ T cell | 7AAD- CD3+ CD4+ |
| Treg cell | 7AAD- CD3+ CD4+ Foxp3+ |
